# Axisymmetrical resonance modes in an electrowetting optical lens

**DOI:** 10.1063/5.0141787

**Published:** 2023-05-17

**Authors:** Eduardo J. Miscles, Wei Yang Lim, Omkar D. Supekar, Mo Zohrabi, Juliet T. Gopinath, Victor M. Bright

**Affiliations:** 1 Department of Mechanical Engineering, University of Colorado, Boulder, Colorado 80309, USA; 2 Department of Electrical, Computer, and Energy Engineering, University of Colorado, Boulder, Colorado 80309, USA; 3 Department of Physics, University of Colorado, Boulder, Colorado 80309, USA; 4 Materials Science and Engineering Program, University of Colorado, Boulder, Colorado 80309, USA

## Abstract

Electrowetting-based adaptive optics are of great interest for applications ranging from confocal microscopy to LIDAR, but the impact of low-frequency mechanical vibration on these devices remains to be studied. We present a simple theoretical model for predicting the resonance modes induced on the liquid interface in conjunction with a numerical simulation. We experimentally confirm the resonance frequencies by contact angle modulation. They are found to be in excellent agreement with the roots of the zero-order Bessel functions of the first kind. Next, we experimentally verify that external axial vibration of an electrowetting lens filled with density mismatched liquids ( Δρ = 250 kg/m^3^) will exhibit observable Bessel modes on the liquid–liquid interface. An electrowetting lens filled with density matched liquids ( Δρ = 4 kg/m^3^) is robust to external axial vibration and is shown to be useful in mitigating the effect of vibrations in an optical system.

Electrowetting-based tunable optics use an applied electric field to manipulate the wettability of a solid surface and modify the contact angle of a liquid droplet with an applied voltage.^1–5^ The relationship is governed by the Lippmann–Young equation,

$$\text{cos}\;(\theta )=\text{cos}\;(\theta_{0})+\eta V^{2},$$ (1)

where *θ* is contact angle due to applied voltage *V*, *θ*_0_ is the zero voltage contact angle, and *η* is the electrowetting constant. Electrowetting optics offer a non-mechanical alternative to tunable lenses, spatial light modulators (SLM), MEMS mirrors, and galvo scanners. Their transmissive nature, lack of mechanical moving parts, low power consumption (*μ*W to mW),^6,7^ range of tunability,^8,9^ and rapid response time^10,11^ have led to implementation in microscopy,^12,13^ miniaturized zoom lenses,^14,15^ microlens arrays,^16,17^ and endoscopes.^18^ Electrowetting prisms have recently been implemented in light detection and ranging (LIDAR),^19^ live animal neuronal imaging,^20^ and wavefront control.^21,22^

While electrowetting-based optics have many attractive features, studying their suitability outside of controlled laboratory environments is of great importance. For example, the robustness of these devices in environments where low-frequency external vibrations caused by air movement of machinery,^23^ aircraft,^24^ or maritime^25^ environments should be characterized. Some studies have implied that devices containing immiscible liquids of equal densities will be less susceptible to vibrations^3,26^ as body forces scale proportionally to the difference in liquid densities. Due to the inherent sensitivity of optical devices, it is important to further understand if density matching is sufficient. Furthermore, a simple model for predicting resonance frequency in an electrowetting device can be used in design to tune the resonance frequencies outside of the operating range. The purpose of the present work is to investigate the resonance modes excited on the liquid–liquid interface of an electrowetting lens device.

Other studies investigating resonance modes on a liquid interface have been primarily focused on inducing forced mixing of fluids in sessile droplets^27–30^ or axially symmetric resonance modes in commercially available electrowetting devices.^31–35^ Electrowetting tunable optics are commonly implemented with two immiscible liquids of contrasting refractive indices in an enclosed cylindrical, conical, or rectangular geometry. The motion of the liquids is constrained by the container walls, and oscillations occur along the liquid–liquid interface. Strauch *et al.* have shown that these oscillations can be modeled as transverse vibrations analogous to a solid circular membrane.^31^ The general time-harmonic solution for interfacial displacement is given by

$$u(r,t)=A\;\text{cos}\;(2\pi ft)J_{0}(\frac{2\pi fr}{c}),$$ (2)

where *A* is the amplitude, *f* is the oscillation frequency, and *J*_0_ is the zeroth order Bessel function of the first kind. From the general time harmonic solution in Eq. (2), the resonance frequencies are determined by imposing boundary conditions on the system. In the case where the liquid meniscus remains immobile at the device radius ( du/dt=0 at *r* = *R*),

$$f_{0n}=\frac{c}{2\pi R}j_{0n},$$ (3)

where $j_{0n}$ is the n^th^ root of the zeroth order Bessel function of the first kind. The capillary wave phase velocity, *c*, can be found by solving Eq. (3) based on the simulated fundamental frequency ( $c=2\pi Rf_{01}/j_{01}$). To measure model accuracy independent of error in device dimensions or liquid properties, we take the ratio between the nth resonance frequency and the fundamental frequency:

$$\frac{f_{0n}}{f_{01}}=\frac{j_{0n}}{j_{01}},$$ (4)

causing the wave velocity and device radius term to drop out.

This study aims to model and predict the resonance frequencies in a cylindrical electrowetting device to inform the design of electrowetting-based optical devices. We investigated the electrowetting lens by modulating the applied voltage and by subjecting the device to external axial vibration. Our theoretical model was expanded to include the effect of different contact angles. We observed that the resonance frequencies corresponded to the roots of the zeroth-order Bessel function of the first kind and that the ratios between higher order frequencies and the fundamental frequency remained consistent regardless of actuation conditions. We also used a simulation to predict the fundamental frequency, which was in good agreement with both the theoretical model and experimental results (∼45 Hz). We then used the simulation to study the response of a density-matched ( Δρ = 4 kg/m^3^) liquid system to external axial vibration and the response of a poorly density-matched ( Δρ = 250 kg/m^3^) liquid system. Finally, we demonstrated that an electrowetting lens filled with density-matched liquids can be used to compensate for vibrations in an optical system when driven with an amplitude-modulated voltage.

To perform frequency domain characterization, a wide range of excitation waveforms can be used as described in Ref. 36. In this study, we use a random-phase multi-sine excitation signal, which is defined by

$$m(t)=\Sigma_{k=1}^{N}\;\text{sin}\;(2\pi f_{k}t+\varphi_{k}),$$ (5)

where *N* is the number of excitation frequencies, *m*(*t*) is the multi-sine excitation signal, and *f_k_* is the modulation frequency. The phase, $\varphi_{k}$, is random values ranging from 0 to 2π. Electrowetting lenses are commonly driven with a high-frequency sinusoidal waveform in order to prevent charge build-up at the triple contact line, which would lead to contact angle relaxation in time.^37^ In this case, the root mean square (RMS) voltage is known to define the contact angle.^9^ The overall driving voltage signal applied to the electrowetting lens is described by

$$V(t)=(A_{c}+m(t))\;\text{sin}\;(2\pi f_{c}t),$$ (6)

where *A_c_* is the carrier amplitude (dictated by desired contact angle) and *f_c_* is the carrier frequency. In this work, *f_c_* = 3000 Hz.^9^

A simulation using COMSOL Multiphysics 6.1 was performed to predict the first resonance frequency and calculate the capillary wave phase velocity of a liquid–liquid interface in an electrowetting device using the Navier–Stokes equation and the finite element method.^8,10,21,38^ The simulation used the Lippmann–Young equation to modulate the contact angle. Using the Lippmann–Young equation to define the contact angle here is a simplification, as the true dynamic contact angle for AC actuation includes a friction term, requiring empirically found friction coefficients.^28,39,40^ We neglect the friction term in our simulation as it has little effect on the shape of standing waves formed on the interface.^35^ The geometry and boundary conditions can be seen in Fig. 1(a). The meniscus was modulated with a random phase multi-sine input voltage at frequencies from 1 to 200 Hz. The displacement of the center point of the liquid–liquid interface was calculated as ΔZ(t), and the frequency spectrum of the center displacement was used to determine the resonance frequency; the spectrum is shown in Fig. 1(b) (dashed line). The fundamental frequency was found to be 45 Hz, and Eq. (4) is used to calculate the capillary wave phase velocity, which is found to be 235  mm/s. A second simulation was performed to study the electrowetting device's response to external axial vibration, using a fixed contact angle and an applied body force. The center displacement was measured, and the frequency spectrum was used to detect resonance modes. The resonance frequency was invariant whether the excitation was an external axial vibration or contact angle modulation, but the amplitude of vibration was much larger for contact angle modulation, as seen in Fig. 1(b). For a system with de-ionized (DI) water and 1-phenyl-1-cyclohexane (PCH), the simulation predicted that 1 g of external axial vibration would cause 25 nm of displacement at the first resonance frequency (*f*_01_). The density and viscosity of liquids used in the simulation are 998 and 994 kg/m^3^ and 1 and 3.26 mPa * s for the DI water and PCH, respectively, with an interfacial surface tension of 16 mN/m.

**FIG. 1.**
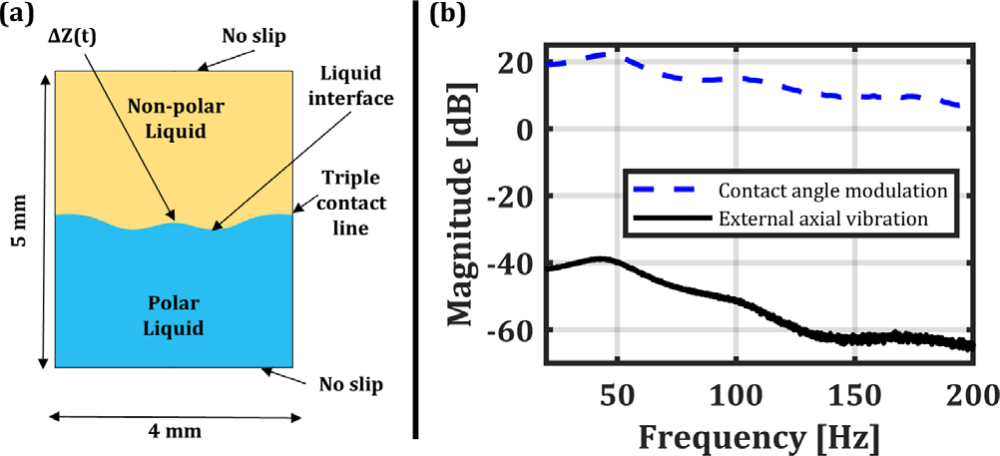
(a) Schematic of the simulation geometry. A 2D axisymmetric condition is used with a Navier slip boundary condition applied to the wall at the contact line. A no slip condition is used on the top and bottom boundaries. The polar liquid is either DI water or SDS dissolved in water, and the non-polar liquid is either PCH or dodecane. The displacement at the center point, ΔZ, is monitored as a function of time. (b) Simulated frequency response of the center displacement when the contact angle is electrically modulated using the multi-sine excitation (dashed) and when the geometry is subjected to an external body force in the z-direction (solid). The three observable peaks in the spectra are in excellent agreement, at 45, 98, and 162 Hz. The magnitude of displacement caused by contact angle modulation is several orders of magnitude larger than that caused by the external axial vibration.

A verification of simulation results is performed with a scaling analysis. When the Bond number, a unitless ratio of body forces to surface tension is much smaller than unity, the interfacial shape is predicted to be a spherical cap.^41^ The Bond number is defined as

$$Bo=\frac{\Delta \rho gR^{2}}{\gamma },$$ (7)

where Δρ is the density mismatch between liquids, *g* is the acceleration due to gravity, and *γ* is the interfacial surface tension. For the DI-PCH liquid combination, the Bond number is 0.009 with the chosen geometry and approaches unity as the applied acceleration approaches 156 g, due to the small density mismatch ( Δρ = 4 kg/m^3^). This agrees with the simulation results, predicting that for accelerations much less than 156 g, the interfacial shape can be accurately represented by a truncated spherical cap. For comparison, a liquid system enclosed in the same geometry consisting of 1 wt. % sodium dodecyl sulfate (SDS) dissolved in water as the polar liquid and dodecane as the non-polar liquid has a Bond number of 1.2, arising primarily from the larger density mismatch ( Δρ = 250 kg/m^3^) as well as lower interfacial surface tension value (*γ*  =  8 mN/m). A simulation of an SDS-dodecane filled electrowetting device predicts ∼4 *μ*m of displacement at resonance when subject to one g sinusoidal axial vibration.

In order to validate both the theoretical model and simulation results, a 4 mm aperture electrowetting lens is used to study the resonance modes at a liquid interface by monitoring light passing through it. The lens is constructed using the same process as Refs. 9, 42, and 43 and is filled with the DI water and PCH liquid combination. A collimated beam passing through the electrowetting lens diverges when the liquid–liquid interface is unactuated but converges when a voltage above 75 V_*rms*_ (90° contact angle) is applied. The light source used is a single mode 635 nm laser collimated to ∼1 mm FWHM, and a 10x long working distance microscope objective is used to collect the diverging beam. A 50 *μ*m pinhole is placed in front of the photodetector (Thorlabs DET36A) to spatially filter the transmitted beam, and the distance between the photodetector and the microscope objective is adjusted to maximize the signal-to-noise ratio. The experimental setup is shown in Fig. 2. The device is driven with an amplitude modulated sinusoidal voltage function, with a 3 kHz carrier signal applied to bias the modulation signal around a specified contact angle. The random-phase multi-sine modulation amplitude is adjusted between 1 and 5 V (RMS) based on the bias voltage. Oscillations of the liquid meniscus along the optical axis are indirectly measured as fluctuations in the transmitted power on the photodetector.

**FIG. 2.**
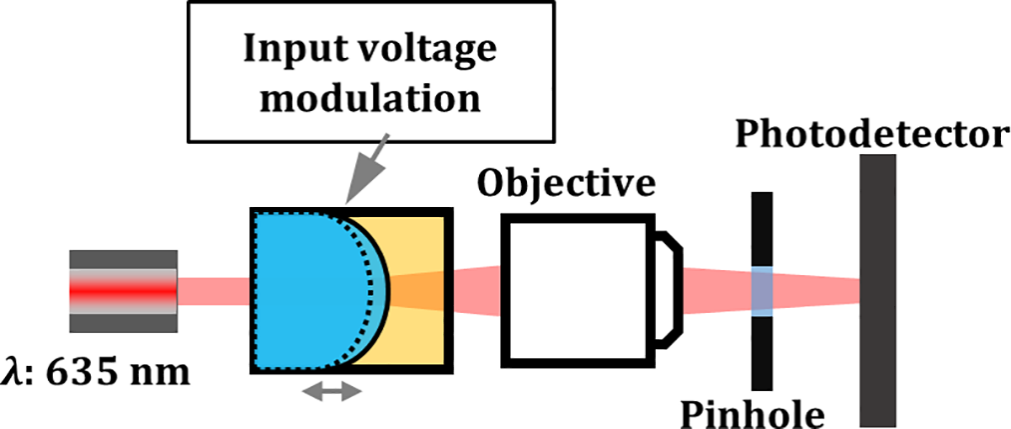
Experimental setup for measuring the resonance modes along the liquid–liquid interface: a laser passes through the center of the 4 mm aperture electrowetting lens device, followed by an objective lens, a 50 *μ*m pinhole to spatially filter the transmitted beam, and a photodetector to collect the transmission signal. The position of the photodetector is adjusted at each biased voltage to compensate for the focal distance shift. Oscillations along the liquid meniscus, ∼1 mm radially around the optical axis, are measured indirectly as a function of transmitted optical power. This optical system is slightly modified and placed on a shaker table for experiments involving applied axial vibration.

The steady-state response is analyzed using a fast Fourier transform (FFT). Gaussian functions are fit to the spectrum to extract the frequency and width of the resonance modes. The Gaussian functions are centered at the lowest 6 resonance frequencies predicted corresponding to the zeroth and first order Bessel modes. While this work focuses on resonances corresponding to the zero-order Bessel modes, Ref. 34 notes that other modes are supported by the interface. We find the zero-order modes to be the dominant modes, as evidenced by the close agreement between theoretical resonances based on the zero-order Bessel modes and experimental resonance. The theoretical, experimental, and simulated resonances shown along with the frequency spectrum biased around 75 V (90° contact angle) in Fig. 3(a), can be seen to be in good agreement. The overall error is attributed to discrepancies in liquid properties, such as viscosity, density, and surface tension used in simulation. The simulation could also be refined by including the effect of the dynamic contact angle such as in Refs. 28, 39, and 40. Furthermore, broadening and reduction in amplitude of higher order modes make the error larger at higher resonance frequencies.

**FIG. 3.**
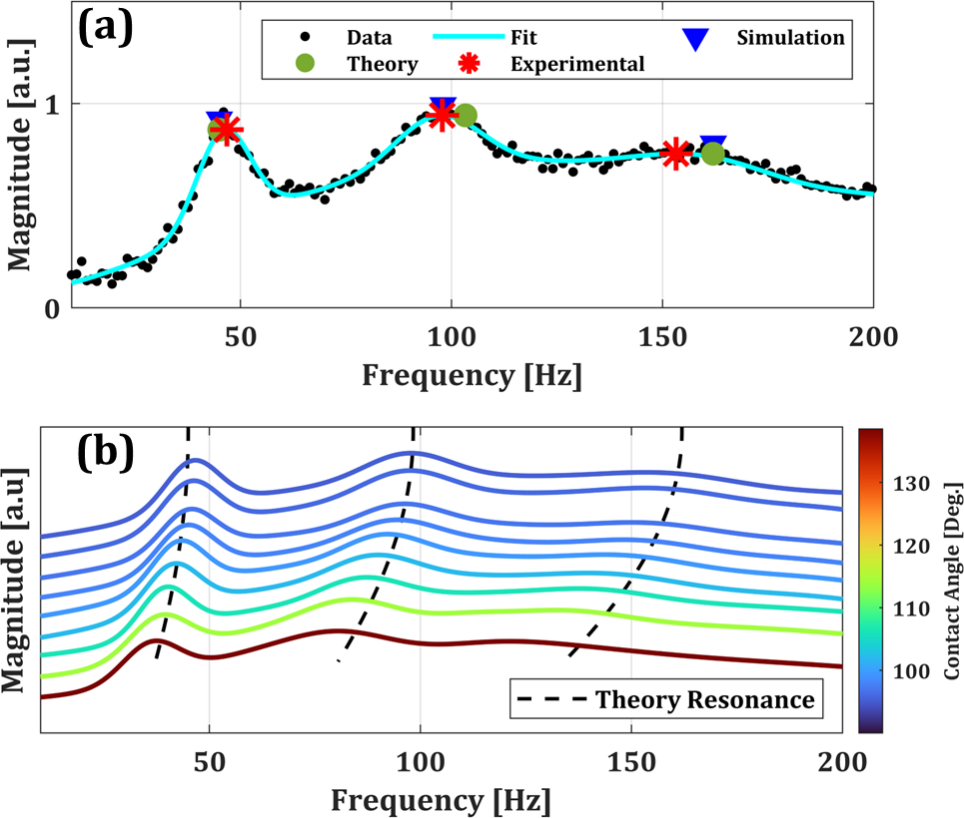
(a) Frequency response of electrowetting lens at a 90° contact angle to an amplitude modulated voltage input. Measured resonance frequencies are labeled with red stars, theoretical predicted resonances are labeled with green circles, and simulated resonance frequencies are denoted by blue triangles. (b) Fits of the experimental transmission spectrum data at varying contact angles, ranging from 138.5° to 90°. Theoretical calculated resonance frequencies are labeled with dashed lines and show good agreement with the peaks in the spectra, having a mean percent error of 6.2%. The second and third resonance frequencies are ∼2.1 and 3.3 times the fundamental frequency, respectively, across the various contact angles.

Figure 3(b) shows the frequency spectra measured for a range of contact angles from 90° to 138.5°. It is clear that as contact angle increases, the resonance frequency decreases. The fundamental frequency ranges from 46.8 Hz for a flat liquid–liquid interface to 37.6 Hz for the largest contact angle measured (138.5°). This is explained by an increase in effective radius, *r_eff_*, over which the capillary wave propagates. For a flat liquid–liquid interface, the distance traveled is equal to the geometric radius of the device, *R*. When the contact angle is higher than 90°, the arc length of the liquid interface becomes the distance traveled. An expression for effective radius is used in conjunction with Eq. (3) for calculation of theoretical resonance

$$r_{\text{eff}}=\frac{\theta -\pi /2}{\;\text{sin}\;(\theta -\pi /2)}R,$$ (8)

where *θ* is the contact angle.

Notably, while the numerical values of the resonance frequencies vary with contact angle, the ratios between the higher order resonance modes and the fundamental mode remain fixed, within measurement error, having a mean value of ∼2.1 and 3.3 for $f_{02}/f_{01}$ and $f_{03}/f_{01}$, respectively, matching the zeroth order Bessel solutions, within 8.5% error. Repeated experiments across electrowetting devices yield consistent results despite minor deviations introduced by device fabrication. The resonance frequencies and their ratios measured for contact angles ranging from 90° to 138° can be found in Table I.

**TABLE I.** Resonance frequency measured on the cylindrical electrowetting device. Bessel ratios between the second order and fundamental frequency are 2.12 ± 0.02, while the third order and fundamental frequency ratio is 3.30 ± 0.05. The measured frequencies and ratios are below 8.5% from the theoretical values.

	Resonance frequencies (Hz)			Frequency ratio	
Contact Angle (°)	*f*_01_	*f*_02_	*f*_03_	$f_{02}/f_{01}$	$f_{03}/f_{01}$
138	37.6	80.6	121	2.14	3.22
133	39.8	83.4	131.2	2.10	3.30
128	40.4	87.4	137.4	2.16	3.40
123	42	90	139.2	2.14	3.31
118	43.4	92.2	143.6	2.12	3.31
114	45.2	94.4	147.8	2.09	3.27
104	45.8	96.2	149.8	2.10	3.27
99	46.2	97.4	153	2.10	3.31
90	46.8	97.8	153.6	2.09	3.28
Percent error	2.9%	7.5%	8.2%	7.8%	8.4%

The presence of Bessel modes when a device is actuated with an amplitude modulated signal have been reported previously in Ref. 31. We extend this by confirming the presence of Bessel modes in an electrowetting lens subject to an external axial vibration. The optical system shown in Fig. 2 is modified by replacing the aperture and photodetector with a fast frame rate camera (Basler acA640-750uc) and using a 60x objective to amplify the light patterns on the camera. The optical system is then vibrated axially on a shaker table. The small oscillation amplitude (25 nm as predicted by the simulation earlier) caused by external axial vibration in the DI water and PCH liquid system, made any induced Bessel modes not visible. Instead, we used the SDS water and dodecane liquid system, as the density mismatch would amplify the interface displacement when the device is subject to external vibration. The induced interfacial Bessel modes are shown in Fig. 4(b). These figures are created by measuring the intensity of the image. The intensity profile confirms the predicted mode shape and the presence of induced Bessel modes on the liquid–liquid interface validates the membrane equivalent model for resonance modes in an electrowetting lens device. We also demonstrated that a well density-matched liquid combination in an electrowetting lens device is robust to external axial vibration in the 1–200 Hz range. An experiment using the electrowetting lens to compensate for vibration in an optical system was also conducted. To measure the transmitted optical power variation in an axially vibrating optical system, we placed the system shown in Fig. 2 on a shaker table and mechanically excited it using a random-phase multi-sine function to characterize its response to vibration in the 1–200 Hz range. We found the system's resonance to be at 55 Hz and observed that it was invariant when an electrowetting lens filled with density-matched liquids (DI water and PCH) was removed and reintroduced. By modulating the device's driving electrical signal with a *π* phase difference to the applied acceleration at 55 Hz, we were able to attenuate the intensity variation of the transmitted beam caused by external axial vibration (1 g) by a factor of ∼4 (Fig. 5). Some oscillations still remained in the compensated waveform, which are attributed to lateral vibration induced in the system by the shaker table, which the lens device was unable to compensate for. The RMS value of normalized intensity variation was reduced from 0.571 to 0.114.

We identified resonance modes in a cylindrical electrowetting device by modulating the liquid–liquid interface at frequencies between 0.1 and 200 Hz and found the fundamental resonance mode shifts toward a higher frequency with increasing contact angle due to increased propagation length from the liquid curvature. Higher order modes followed the roots of the zeroth order Bessel function of the first kind. A time-dependent simulation approach was able to predict the fundamental frequency, and the theoretical model predicted resonance frequencies within 8.5% deviation. Our findings demonstrate that a well density-matched liquid combination is robust to low-frequency external vibrations, making electrowetting optics suitable for use in live animal imaging, LIDAR, and machine vision imaging systems. These findings also show promise for applications, such as interface shape control, active optical system vibration compensation, and interfacial wave cancelation for novel liquid combinations.

**FIG. 4.**
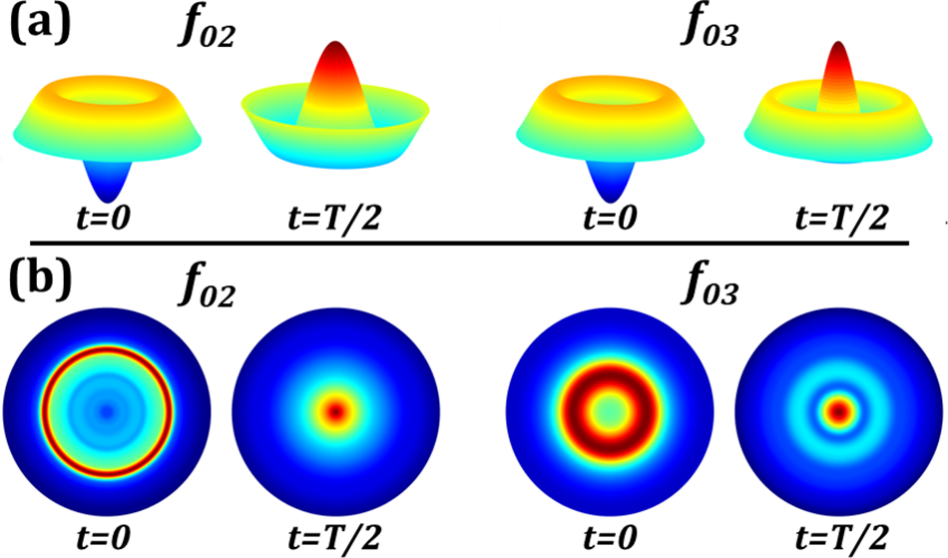
(a) Predicted interfacial shape at resonance corresponding to the roots of the zeroth-order Bessel function. (b) Intensity map of a beam transmitted through a vibrating liquid–liquid interface in an electrowetting lens. The vibration acceleration ranges from 4 to 9 g to enhance the oscillation profile. The first mode is not shown as it only causes focus/defocus. These are created by taking the linear intensity profile along the center, and mapping to a circular geometry.

**FIG. 5.**
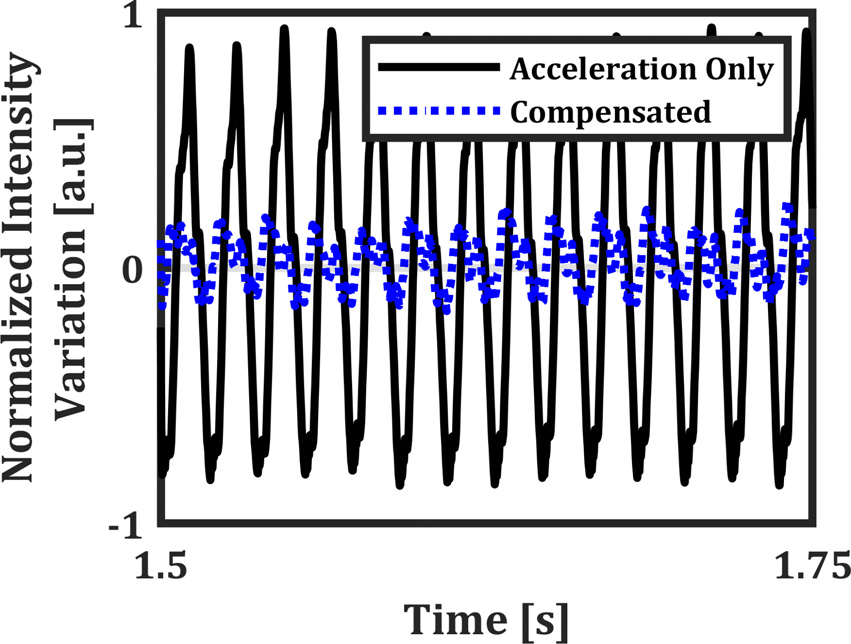
Steady-state response to 1 g external axial vibration at 55 Hz. Black shows response to vibration only, while blue shows the suppressed response by using an amplitude modulated lens driving signal. The RMS value of normalized intensity variation is reduced from 0.571 to 0.114.

## Data Availability

The data that support the findings of this study are available from the corresponding author upon reasonable request.
